# Supplementary material for: Organization of the Catecholaminergic System in the Short-Lived Fish Nothobranchius furzeri
Source: Front Neuroanat. 2021 Sep 13;15:728720. doi: 10.3389/fnana.2021.728720 (PMC8473916; doi:10.3389/fnana.2021.728720)
Supplement: Supplementary file 1 [file Data_Sheet_1.pdf]

Supplementary Material

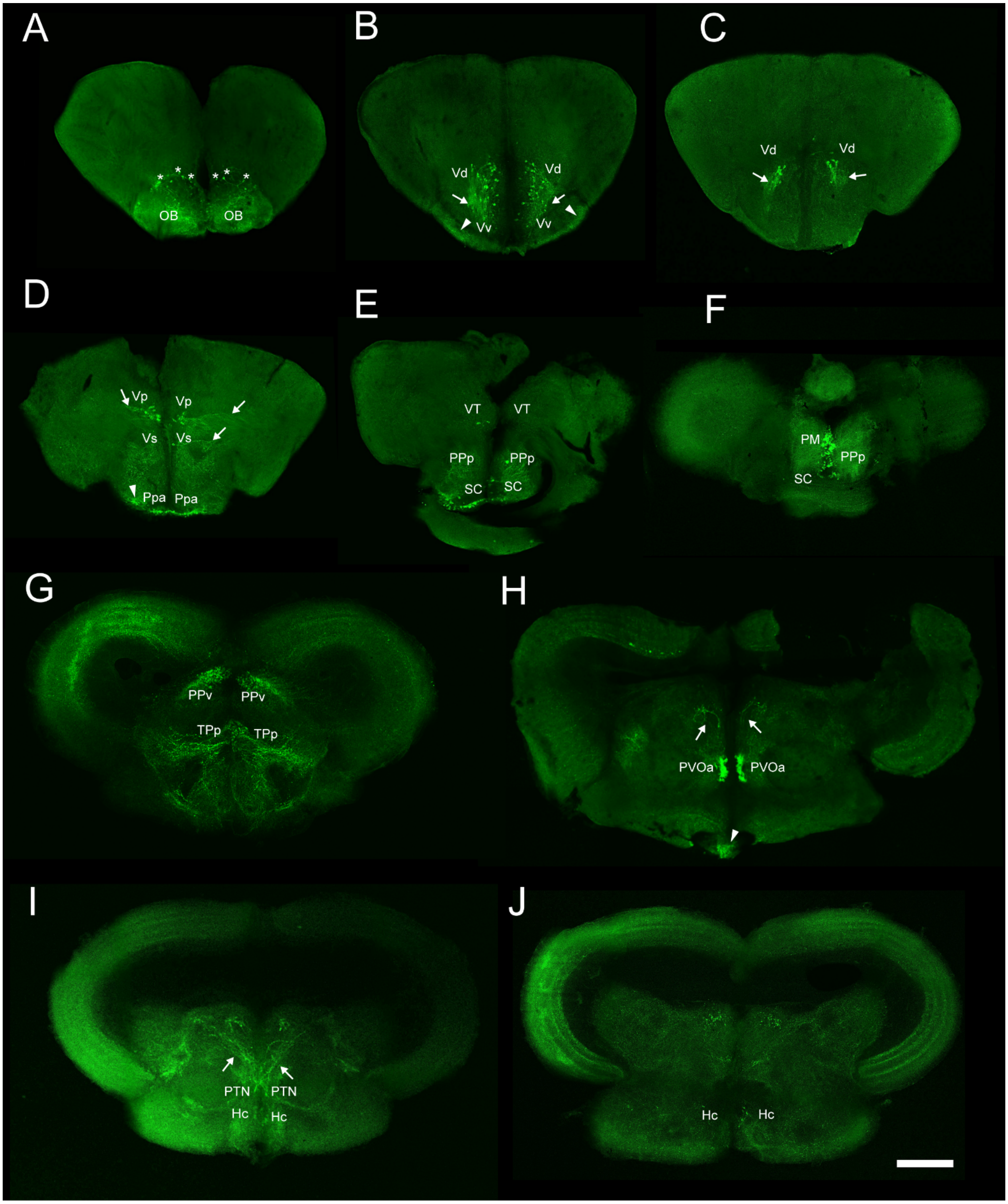

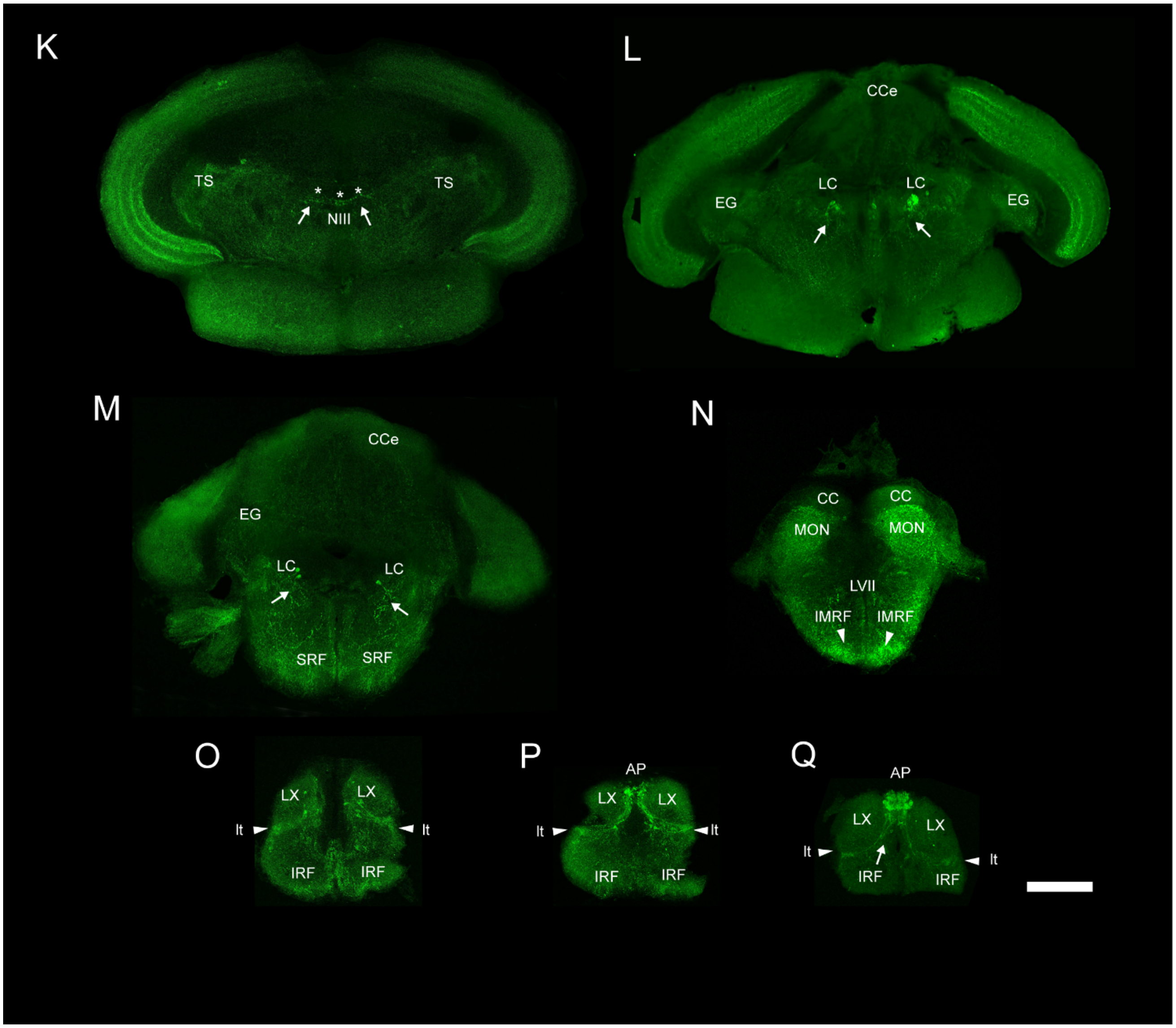

Supplementary Figure 1. Photomicrographs of transverse sections (in rostral to caudal order) showing the anti-TH immunofluorescence in the brain of *Nothobranchius furzeri*. Asterisks point neuronal somas, arrows show the direction of the processes and arrowheads indicate regions with intense density of TH+ neuropil or tracts. For more details see the text and for abbreviations see list. Scale bar, 500 µm.

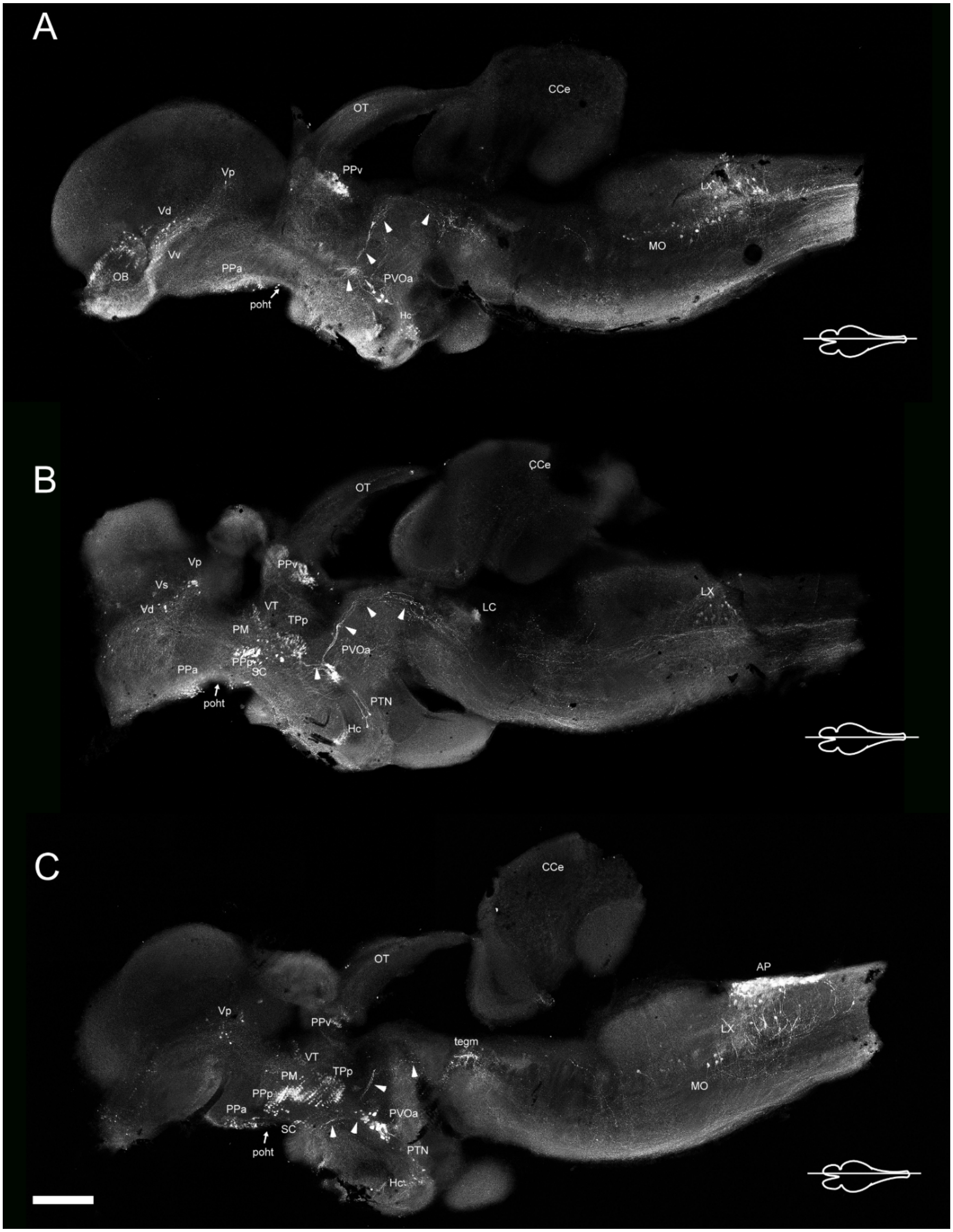

Supplementary Figure 2. Photomicrographs of para-sagittal sections near the midline (from lateral towards the midline), showing the distribution of TH+ neuronal groups and fibers/tracts through the brain of *Nothobranchius furzeri*. Arrowheads point to TH+ fibers arising from the paraventricular organ-accompanying cells and arrows indicate the preopticohypothalamic tract. For more details see the text and for abbreviations see list. Scale bar, 500 µm.

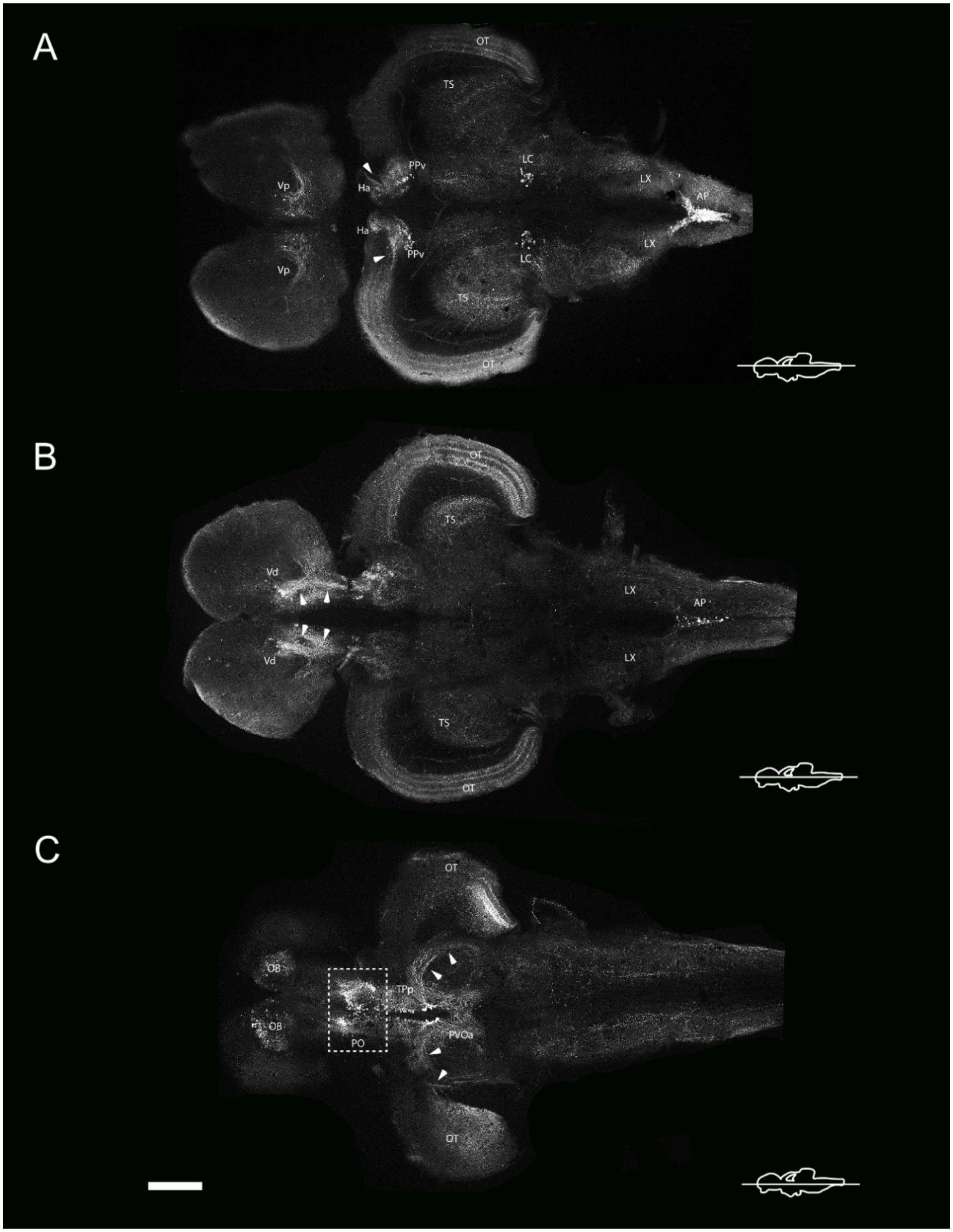

Supplementary Figure 3. Photomicrographs of horizontal sections (in dorsal to ventral order) showing the distribution of TH+ cellular groups and fibers/tracts along the rostrocaudal axis of the brain in *Nothobranchius furzeri*. Arrowheads in **(A)** indicate the TH+ fibers of the ventral periventricular pretectal nucleus reaching the optic tectum, in **(B)** the fibers from the posterior zone of ventral telencephalon following a caudal direction, and in **(C)** the direction of processes from the paraventricular organ-accompanying cells. For more details see the text and for abbreviations see list. Scale bar, 500 µm
